# Nature and extent of outpatient podiatry service utilisation in people with diabetes undergoing minor foot amputations: a retrospective clinical audit

**DOI:** 10.1186/s13047-020-00445-5

**Published:** 2021-01-13

**Authors:** Clare Linton, Angela Searle, Fiona Hawke, Peta Ellen Tehan, Vivienne Chuter

**Affiliations:** 1 BE130 Health Precinct, Brush Road, Ourimbah, NSW 2258 Australia; 2 Central Coast Local Health District, Gosford Hospital, Holden St, Gosford, NSW Australia; 3 School of Health Sciences, Faculty of Health and Medicine, The University of Newcastle, Ourimbah, NSW Australia

**Keywords:** Minor foot amputation, Diabetes, Podiatry, High risk foot clinic

## Abstract

**Background:**

People with diabetes are at high risk of foot complications that can lead to lower extremity amputations. National standards suggest that early assessment and management by a podiatry led multidisciplinary high-risk foot clinic (HRFC) helps to reduce complications. This review is a retrospective audit of the Central Coast Local Health District (CCLHD) podiatry department service utilisation in people with diabetes who had undergone a minor foot amputation.

**Methods:**

All people with diabetes who had minor foot amputations in the calendar year 2017 in the CCLHD in New South Wales were identified. Podiatry occasions of service from all podiatry service clinics (e.g. general, orthoses, wound, HRFC) and hospital stays for 12 months prior to, and 12 months, post the minor foot amputation were extracted.

**Results:**

Data on 74 people with diabetes who underwent 85 minor foot amputations were collected. In the 12-month period leading up to their minor foot amputation less than half, 42% (*n*=31), of the patients had attended any of the available podiatry service clinics within the CCLHD system. Post-amputation and discharge from hospital there was an overall rise of 26% in numbers attending all CCLHD podiatry- led clinics bringing the total to 68% (51). However, attendance at the HRFC rose by only 2% from 16% (*n*=12) to 18% n= (13).

**Conclusion:**

This study shows that there was underutilisation of Podiatry Services in the CCLHD in 2017 with some participants not meeting national treatment guidelines for foot health services. Revision of current referral pathways both prior to, during and following hospitalisation and expanding the multidisciplinary HRFC to accommodate the population by providing more accessible locations has since been undertaken to increase service access. Further provision of education to those highlighted to be at high risk has also been implemented.

## Background

Current guidelines for the management of diabetes-related foot disease recommend a multidisciplinary team approach to clinical assessment, treatment, and management of contributing foot complications [1–4]. Reports state that effective multidisciplinary interventions have the potential to reduce complications, assist in prevention and decrease the frequency and severity of diabetes-related amputations [1, 2].

Podiatrists have a key role in these multidisciplinary teams as they deal with the prevention, diagnosis and treatment of foot and lower leg conditions, including the management of the diabetic foot [5, 6]. Early diagnosis of diabetes-related foot complications such as peripheral arterial disease (PAD) and peripheral neuropathy (PN), along with effective preventative care including appropriate footwear and pressure offloading, reduces risk of diabetic foot ulceration (DFU) and amputation, thereby preserving an individual’s mobility and independence and reducing health care costs [5, 7–9].

Podiatry care post-amputation is critical. There is strong evidence that people with diabetes who are hospitalised for foot-related disease are at high risk of further foot complications [10]. This is particularly the case with those undergoing minor foot amputations where there are high rates of post-surgical delayed healing, infection and risk of more proximal amputation [11, 12]. In these circumstances access to a multidisciplinary foot care clinic may improve healing outcomes [13].

The evaluation of data, gathered from both hospital and community care, of people with diabetes undergoing minor amputation will assist in evaluating current care provision throughout the entire patient journey and direct strategy to improve health outcomes. This is especially critical for the Central Coast Local Health District (CCLHD) as this district has the second highest rate of diabetes in New South Wales (NSW) and the numbers are rising [14]. Therefore, the aim of this audit was to examine the nature and extent of podiatry service utilisation 12 months pre and post minor diabetes-related foot amputation in the 2017 calendar year, as part of an ongoing quality improvement process.

## Methods

### Design

Ethics approval for this project was provided by the Hunter New England Research Ethics & Governance Office (2019/ETH10678) and the Central Coast Local Health District (CCLHD) Site Specific Assessment (SSA) approval (2019/STE13574). All people with diabetes in the CCLHD who underwent minor foot amputations within the calendar year of 2017 were identified. Hospital stay and podiatry utilisation records for the people undergoing these amputations were extracted for a period of 12 months either side of the amputation.

### Participants

The CCLHD is located on the east coast of NSW between Sydney and Newcastle. It has a population of around 300,000 and in 2017 was divided into Gosford and Wyong shires with Wyong displaying a higher than average population growth [15]. The population of the Central Coast of NSW in 2016 was 327,736 and the Wyong area ranked four out of ten in the Socio-Economic Index for Area (SEIFA) rating scale placing it in the low range [16]. Overall, the area had a workforce rating of five out of ten and a high manual labour workforce [16] which can be correlated with lower income. The area is serviced by two acute hospitals, Gosford and Wyong, which had, in 2017, an acute inpatient bed capacity of 368 and 182 respectively [14]. Public podiatry outpatient services are attached to both hospitals. Eligibility criteria for assessment and general foot care at the podiatry outpatient services are restricted to those with diagnosed diabetes and on a current pension or health care card, or anyone with wounds below the malleoli. The Services offer general foot care and assessment clinics, orthoses (biomechanical) clinics and a high-risk foot clinic (HRFC). The aim of the HRFC is to provide a multidisciplinary team that is highly skilled and works together in a coordinated approach to target those at most risk and achieve best outcomes for the attending clients. In 2017 the HRFC was only available at one location (Wyong) on a fortnightly basis and consisted of a Vascular specialist and a podiatry team, while further health specialities and disciplines were available on a referral basis.

### Criteria

Inclusion criteria for this study was limited to people with minor foot amputations, minor amputations were classified as an amputation distal to the ankle joint as specified by Nather et al. [17], with any type of diabetes, within either Gosford or Wyong hospitals during the period of 1st January 2017 until 31st December 2017. Amputations related to a cancer related illness or a major trauma, such as a motor vehicle accident were excluded.

### Data collection

Both hospital inpatient and community outpatient data were extracted for the included patients. Inpatient data were collected on 13.11.2019 via access to the electronic medical record system (eMR). Specific point-of-care data was collected for the 12 months prior to amputation and for the stay of hospitalisation. Demographic data and medical history including diabetes type, oral hypoglycaemic/insulin usage, history of foot ulceration, history of tobacco use, cardiovascular disease, renal disease, lower limb infection and ischaemia with and without gangrene were extracted.

Outpatient data were sourced from ComCare, a community-based data collection programme that stores information in a centralised and integrated software system. This encompassed any occasions of service (OOS) from podiatry and multi-disciplinary teams for the 12 months preceding the amputation and the 12 months following discharge from hospital. Podiatry specific data were extracted from ComCare, including type of service (wound clinic, HRFC, general clinic, biomechanical clinic) and number of OOS (including did not attend) for the same period.

### Statistics

All statistical tests were conducted using SPSS Release 24 for Windows (SPSS Inc., Chicago, Ill., USA). Differences in patient characteristics between pre and post amputation podiatry client groups were evaluated by independent samples t-test for continuous variables and Chi-square test for categorical variables [18]. Statistical significance was delimited at *p* < 0.05.

## Results

**Figure 1:**
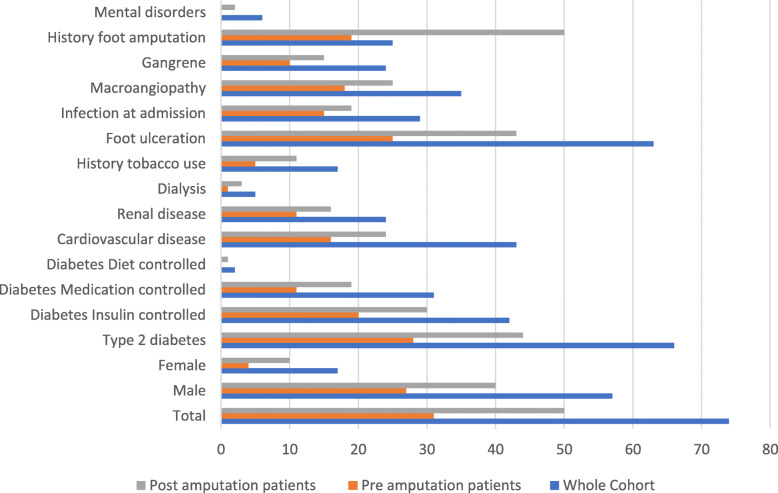
Characteristics of the study population. Pre and Post amputation numbers do not include patients who attended only a single appointment prior to (*n*=2) or post (*n*=1) their amputation

Seventy-four people with diabetes who underwent eighty-five minor foot amputations in the calendar year 2017 in the CCLHD were identified by the audit. Fifty-eight (78%) of the people were aged 60 years and over, and fifty-seven (77%) were male (Fig. 1). Of the patients, 85% (*n*= 63), presented with an existing foot ulceration and 39% (*n*=29) had a current infection at the time of admission (Fig. 1). A third of the people, 34% (*n*= 25), had a history of previous foot amputations (Fig. 1).

### Podiatry patients and utilisation

In the 12-month period leading up to their minor foot amputation less than half, 42% (*n*=31), of the study patients had attended any of the available clinics within the CCLHD Podiatry Services (Table 1.). There was a low attendance rate at the multidisciplinary podiatry led HRFC prior to amputation of 16% (*n*=12) people and 15% (*n*=11) of patients failed to attend a booked appointment in any clinic. Significantly more of the people who attended the Podiatry Services prior to their amputation reported a history of previous minor foot amputations (76% vs 24%), and this group also had significantly less mental disorders (including dementia and schizophrenia) (0% vs 6%) than those who did not use the clinics (Fig. 1).

**Table 1:** CCLHD podiatry clinic usage. Values are number (%) unless stated otherwise

	Pre amputation		During admission	Post amputation	
	Clinic attendance (people)	Median visits		Clinic attendance (people)	Median visits
**Overall**	31 (42%)	14	13 (18%)	50 (68%)	15
**HRFC**	12 (16%)	5		13 (18%)	6
**Failed to attend**	11 (15%)	1		16 (22%)	1.5
**Wound clinics**	28 (38%)	10		44 (60%)	9
**Orthoses clinics**	1 (1%)	3		13 (18%)	2

*Abbreviations*: *CCLHD* Central Coast Local Health District, *HRFC* High Risk Foot Clinic

During the hospital stay podiatry interventions were administered to thirteen (18%) patients (Table 1). Following discharge from hospital twenty-five patients were referred or re-referred back to the CCLHD Podiatry Services. Two referrals (8%) were via medical specialists, seven referrals (28%) were part of the hospital discharge plan, five referrals (20%) were directly from the patient’s general practitioner and eleven referrals (44%) were from the CCLHD community nurses, who are encouraged to refer foot wounds back to CCLHD Podiatry Services for specialised care.

Overall, there was a rise of 26% in numbers or patients attending any CCLHD podiatry-led clinics post amputation bringing the total to 68% (50), excluding one person who attended only one clinic. Attendance at the HRFC had the smallest increase of 2 to 18% (13), whereas wound clinic attendance rose from 38 to 60% (*n*=44). Non-attendance at the clinics also increased, by 7 to 22% (*n*=16) (Table 1).

## Discussion

This audit highlights several pathways within the existing provision of CCLHD Podiatry Services that will add improvements to service delivery. There is a plethora of guidelines [2, 4, 10, 19–22] that recommend people with diabetes attend, at minimum, an annual foot assessment. This assessment provides an opportunity to highlight, treat and monitor areas or issues that may become problematic and provide timely interventions to reduce the risk of ulceration, infection and potential amputation [3, 23–26]. The International Working Group for the Diabetic Foot (IWGDF) found that for people with diabetes and those at high risk of complications, annual attendance at a multidisciplinary foot care clinic resulted in a reduced risk of developing foot ulceration [27]. However, our results show that less than half the people we identified who had a minor foot amputation in 2017 had attended a CCLHD Podiatry clinic in the 12 months prior to their amputation.

Previous research has shown high rates of diabetic foot disease (DFD) in regional and rural populations in Australia with poorer general health outcomes away from major cities and highlights the need for accessible health care [28–30]. Reduced physical accessibility to high risk foot care may be a contributing factor in the low rates of podiatry care access seen in our study cohort. The CCLHD Podiatry department has a small staff profile which operates primarily as a community outpatient service and covers a large geographical area. During the period of the audit wound care clinics were available at both Gosford and Wyong Hospitals but the multidisciplinary HRFC only operated out of a single location at Wyong Hospital, which is situated at the northern end of the district. Overall, 80% of the amputations in this audit occurred in Gosford hospital, with the patients residing within its more southern catchment area. For people in the southern end of the district this could mean a travel distance of up to 61 km to attend the multidisciplinary HRFC. The travel distance, in conjunction with poor public transport requiring multiple changes, could be prohibitive for clients with existing diabetes-related foot conditions. This could account for the low HRFC numbers and the rise in attendance at the more accessible wound clinics following amputation, from 38% (*n*=28) to 60% (*n*=44). While some patients may have been accessing private sector podiatry services these are not typically used for wound care due to excessive costs (31) to the individual.

In regional Victoria DFD has been shown to be disproportionately represented in socio-economically disadvantaged Australians [28]. Research has shown that people in lower socioeconomic groups are at greater risk of illness, such as diabetes, disability and death [31]. Studies also show that people from low socioeconomic areas are more likely to be poorly educated in regard to their physical and psychological health and that many behaviours are passed down through generations and directly affect their lifestyle choices including attending health care appointments [32]. These socioeconomic factors, in conjunction the previously discussed limitations to service access, may have also contributed to the low attendance rates.

Of note, was the discrepancy in the number of females undergoing minor foot amputations in 2017 (23% of the cohort), compared to those who had attended any of the CCLHD Podiatry Services (13% of the cohort). On investigation the females in our cohort were significantly older than the males (<*p*=0.0001) and also significantly more likely to suffer from mental health disorders (28% vs 2%, *p*=0.047) including dementia and schizophrenia. These conditions are likely to have impacted the patients’ ability to attend regular appointments in the CCLHD Podiatry Services.

The New South Wales Standards for High Risk Foot Services document [33], on which the CCLHD benchmark Podiatry Services, contains eleven recommended standards including a multidisciplinary approach, clinical leadership and coordination, administrative support, evidence based treatment guidelines, continuity of care across settings, prompt access for urgent cases, located within a health facility and access to onsite services, appropriate equipment, pressure offloading and provision of medical grade footwear and recording and monitoring of clinical outcomes [34]. Standard five requires a continuity of care across inpatient and outpatient health settings. This was shown to be less than optimal in both settings and is observable in the low number of inpatient podiatry services provided, to only 18% of the clients. Low numbers of patients being assessed by podiatry staff during the hospital stay not only reduces best practice care but also reduces opportunities for education and interventions that could improve wound healing and prevent future ulcerations. Similarly, a minority of the patients (28%) received a referral for outpatient podiatry services as part of the hospital discharge process. It is possible that these results are a consequence of referral pathways that were not adequately formalised or documented, and which are commonly made on the basis of specialist opinion after consideration of individual patient circumstances. Work is now underway to further clarify formalisation of, and adherence to referral options and pathways.

Additionally, the low numbers of clients attending an orthoses clinic further reflects that opportunities to offload areas of high pressure and potentially reduce the risk of foot ulceration are not being met, and that the provision of medical grade footwear is not being achieved. Pre amputation numbers showed only 1% (*n*=1) of clients were accessing the orthoses clinic, and while post amputation attendance did rise, it was only to 18% (*n*=13). People with a history of foot amputation require custom moulded foot orthoses and medical grade footwear [35] to accommodate unique foot shapes and to reduce the high risk of further foot ulceration. Specialised clinics can assess the feet and provide recommendations for custom footwear and orthoses and aid the person in accessing government funding sources.

Further to this audit, the multidisciplinary HRF clinic has been expanded to operate out of both Wyong and Gosford Hospital allowing for easier access to those in the southern portion of the district. Additional health specialities have also been added to every HRFC and now include the services of Endocrinology, diabetes education and dietetics which have adhered to the NSW standards (standard 1) and further advanced the service. Inpatient services have expanded to include podiatry on ward rounds to ensure continuity of care and a better capture of post-operative clients (standard 5). This study highlights the need for organisations to continually monitor and evaluate the health pathways available within their service, to ensure early recognition of those who are at high risk of lower limb amputation, and adequate provision of access to care.

The conclusions in this review must be considered in light of certain limitations. Our study was limited to review of public podiatry services and we have no data regarding people attending private podiatrists, or private hospitals prior to or after their minor foot amputation.

## Conclusion

This study reveals that the CCLHD Podiatry Services were underutilised in 2017, especially in relation to low rates of access prior to minor foot amputations. Following the review, ongoing improvements to services include greater inpatient focus, expanding the multidisciplinary HRFC to provide more accessible locations, providing education for to those highlighted to be at high risk(including daily monitoring of feet, identification of early signs of infection and seeking medical assessment for any foot trauma), and actions to initiate and encourage referral pathways both prior to, during and following hospitalisation. These steps will increase access to the CCLHD Podiatry Services and reduce risk of minor foot amputations.

## Data Availability

The datasets used and/or analysed during the current study are available from the corresponding author on reasonable request.

## Abbreviations

LEA: Lower extremity amputation
HRFCS: High Risk Foot Clinic
CCLHD: Central Coast Local Health District
PN: Peripheral neuropathy
PAD: Peripheral arterial disease
DFU: Diabetic foot ulceration
OOS: Occasions of service
DFD: Diabetic foot disease

## References

[1] National Institute for Health and Care Excellence NICE. Diabetic foot problems: prevention and management. UK: NICE guideline; 2015. 26741017

[2] Schaper NC, van Netten JJ, Apelqvist J, Bus SA, Hinchliffe RJ, Lipsky BA, Schaper NC, Jaap J, Apelqvist J, Bus SA, Robert J, Benjamin H, Lipsky A (2019). IWGDF Guidelines on the prevention and management of diabetic foot disease, in Practical Guidelines, v.N.

[3] Diabetic Foot Australia, Australian and International Guidelines on Diabetic foot disease (2016). IWGDF recomendations on peripheral artery disease and infection.

[4] Heart MBIDI, National Health & Medical Research Council (NHMRC) (2011). National Evidence-based Guideline on prevention ,Identification and Management of Foot Complications in Diabetes in Guideline on Management of Type 2 Diabetes.

[5] Lazzarini PA, O'Rourke SR, Russell AW, Derhy PH, Kamp MC. Reduced incidence of foot-related hospitalisation and amputation amongst persons with diabetes in Queensland, Australia. PLoS One. 2015;10(6). 10.1371/journal.pone.0130609 PMC4476617 26098890

[6] Schaper NC (2019). IWGDF guideline on diagnosis, prognosis and management of peripheral arterial disease in patients with a foot ulcer and diabetes.

[7] Markakis K, Bowling F, Boulton A (2016). The diabetic foot in 2015: an overview. Diabetes Metab Res Rev.

[8] Frykberg RG (2006). Diabetic Foot Disorders. A clinical practice Guidelines (2006 Revision). J Foot Ankle Surg.

[9] Wraight PR, Lawrence SM, Campbell DA, Colman PG. Creation of a multidisciplinary, evidence based, clinical guideline for the assessmant, investigation and management of acute diabetes related foot complications Diabetes UK. Diabet Med. 2004;22:127–36. 10.1111/j.1464-5491.2004.01363.x 15660728

[10] Chadwick PE, McCardle M, Joanne & Armstrong, David, Best Practice Guidelines: Wound Management in Diabetic Foot Ulcers. Wounds International. 2013. p. 1–23. https://www.woundsinternational.com. Accessed 10 Aug 2020.

[11] van Netten JJ, Baba M, Lazzarini PA (2017). Epidemiology of diabetic foot disease and diabetes-related lower-extremity amputation in Australia: A systematic review protocol. Systematic Rev..

[12] Kurowski JR, Nedkoff L, Schoen DE, Knuiman M, Norman PE, Briffa TG (2015). Temporal trends in initial and recurrent lower extremity amputations in people with and without diabetes in Western Australia from 2000 to 2010. Diabetes Res Clin Pract.

[13] Moulik PK, Mtonga R, Gill GV (2003). Amputation and mortality in new-onset diabetic foot ulcers stratified by etiology. Diabetes Care.

[14] NSW Government (2017). Central Coast Local Health District 2017-2022 Clinical Services Plan.

[15] NSW Government (2018). Diabetes prevalence in adults. Health stats NSW.

[16] Australian Bureau of Statistics (2011). SEIFA by Local Government Area (LGA).

[17] Nather A, Wong K (2013). Distal amputations for the diabetic foot. Diabetic Foot Ankle.

[18] Portney L, Watkins M (2009). Foundations of Clinical Research. Applications to Practice., ed. Pearson.

[19] Botros MK, Embil J, Goettl J, Morin K, Parsons C, Scharfstein L, B Somayaji R, Evans R (2017). Best practice recomendations for the Prevention and management of diabetic foot ulcers*.* Foundations of Best Practice for Skin and Wound Management.

[20] Frykberg RG (2006). Diabetic foot disorders. A clinical practice guideline (2006 Revision). J Foot Ankle Surg.

[21] Armstrong D, Lavery LA, Harkless LB (1996). Treatment-based Classification System for Assessment and Care of Diabetic Feet. J Am Podiatr Med Assoc..

[22] Harding K (2016). Local management of Diabetic foot Ulcers in Position Document W.U.o.W.H. Societies.

[23] Diabetes Australia. 4,400 reasons to take diabetes seriously. 2016. Available: https://www.diabetesaustralia.com.au/mediarelease/amputations-4400-reasons-to-take-diabetes-seriously/. Accessed 10 Aug 2020.

[24] Ogrin R, Sands A (2006). Foot assessment in patients with diabetes. Aust Fam Physician.

[25] Quinton T, Lazzarini PA, Boyle FM, Russell AW, Armstrong DG (2015). How do Australian podiatrists manage patients with diabetes? The Australian diabetic foot management survey. J Foot Ankle Res..

[26] van Netten JJ, Lazzarini PAF, Kinnear R, Griffiths E, Malone I, Perrin M, Prentice BM, Sethi J, Wraight S. P R, Australian diabetes related foot diseases strategy 2018–2022: The first step towards ending avoidable amputations within a generation. Brisbane: Diabetic Foot Australia, Wound Management CRC; 2017.

[27] van Netten J, Lavery PE, Monteiro-Soares LA, Rasmussen A, Jubiz Y, Bus SA (2016). Prevention of Foot Ulcers in the At-Risk Patient with Diabetes: A Systematic Review. Diab Metab Syndr Clin Res Rev.

[28] Perrin BM, Gardner MJ, Kennett SR (2012). The foot-health of people with diabetes in a regional Australian population: a prospective clinical audit. J Foot Ankle Res.

[29] Commons RJ, Robinson CH, Gawler D, Davis JS, Price RN (2015). High burden of diabetic foot infections in the top end of Australia: an emerging health crisis (DEFINE study). Diabetes Res Clin Pract.

[30] Bywood P, Lunnay B, K.R (2011). Disparities in primary health care utilisation: Who are the disadvantaged groups? How are they disadvantaged? What interventions work?.

[31] Australian Government. Australian Institute of Health and Welfare. Australia's health 2016. Health across socioeconomic groups 2016 [cited 2020 8th August]; Available from: https://www.aihw.gov.au/getmedia/405d9955-c170-4c39-a496-3839059149f7/ah16-5-1-health-across-socioeconomic-groups.pdf.aspx.

[32] Rasekaba TM, Lim WK, Hutchinson AF (2012). Effect of a chronic disease management service for patients with diabetes on hospitalisation and acute care costs. Aust Health Rev.

[33] NSW Agency for Clinical Innovation ACI, Standards for High Risk Foot Services (HRFS) in NSW (2014). Standards in Health Care.

[34] Innovation, N.A.f.C. Standards for High Risk Foot Services (HRFS) in NSW, Agency for Clinical Innovation. Chatswood; 2014.

[35] Bergin SM, Nube VL, Alforf JB, Allard BP, Gurr JM, Holland EL, Horsley MW, Kamp MC, Lazzarini PA, Sinha AK, Warnock JT (2013). Australian diabetes foot network: practical guideline on the provision of footwear for people with diabetes. J Foot Ankle Res.

